# Improving the Knowledge on Distribution, Food Preferences and DNA Barcoding of Natura 2000 Protected Species *Paracossulus thrips* (Lepidoptera, Cossidae) in Romania

**DOI:** 10.3390/insects12121087

**Published:** 2021-12-03

**Authors:** Geanina Magdalena Iacob, Cristina Craioveanu, Vladimír Hula, Virgiliu Marius Aurelian, Monica Beldean, Cristian Sitar

**Affiliations:** 1 Sociobiology and Insect Ecology Lab, Department of Taxonomy and Ecology, Babes-Bolyai University, Clinicilor 5, 400006 Cluj-Napoca, Romania; giacob@yahoo.com (G.M.I.); cristinacraioveanu@gmail.com (C.C.); marius.v.aurelian@gmail.com (V.M.A.); monicabeldean@gmail.com (M.B.); 2 Department of Forest Ecology, Faculty of Forestry and Wood Technology, Mendel University in Brno, 613 00 Brno, Czech Republic; Hula@mendelu.cz; 3 Asociația Pentru Natura și Oameni, 407240 Cojocna, Romania; 4 Zoological Museum, Babeș Bolyai University, Clinicilor 5, 400006 Cluj-Napoca, Romania; 5 Romanian Institute of Science and Technology, Saturn 24-26, 400504 Cluj-Napoca, Romania

**Keywords:** *Paracossulus trips*, *Phlomis tuberosa*, habitat, biology, ecology, conservation, CO1

## Abstract

**Simple Summary:**

NATURA 2000 species, *Paracossulus thrips* is one of the locally distributed, least studied moth species and is currently considered an endangered species in several European countries, even though the risk factors and its biology and ecology are not well known. In our study, conducted in Transylvania, Romania, we confirm the host plant as *Phlomis tuberosa* and describe the habitat type of *P. thrips*; we also present four new populations and the first DNA barcoding sequences for the species. Our study provides baseline knowledge on the biology and ecology of *P.thrips,* relevant for conservation, and for establishing management measures.

**Abstract:**

*Paracossulus thrips* (Lepidoptera, Cossidae) is one of the locally distributed and endangered species. In Europe, it is also one of the few protected moth species, through Annexes II and IV of the Council Directive 92/43/EEC, Annex II of the Bern Convention. To date, little is known about the biology and ecology of this species. Our study was conducted in Transylvania, Romania. Romania hosts some of the strongest populations of the species in the European region. As part of the study, we conducted field observations, vegetation analyses, and genetic analyses. In our paper, we show the habitat types where we encounter *P. thrips* in Transylvania and confirm *Phlomis tuberosa* as a host plant. Furthermore, a piece of important information for habitat conservation is given. In this paper, we present the eggs and larvae of *P. thrips*, the first DNA barcoding sequences, and four new populations of *P. thrips* in Romania. Our study provides baseline knowledge about the biology and ecology of *P. thrips*, which is important for conservation and establishing management measures.

## 1. Introduction

*Paracossulus thrips* (Hübner, 1818) (Lepidoptera, Cossidae) is a locally distributed, poorly studied moth species. It is currently considered an endangered species in several European countries, although the risk factors are not well known. In Europe, it is also one of the few protected moth species, through Annexes II and IV of the Council Directive 92/43/EEC [1,2], Annex II of the Bern Convention and, not evaluated species in IUCN Red List. In the Romanian Red List of Lepidoptera, it is marked as vulnerable (VU) [3]. In the Bulgarian Red List it is critically endangered (CR) [2].

However, very little is known about the biology and ecology of *P. thrips*. Most studies are limited to reporting the presence of the species and providing a general description of the habitat.

*P. thrips* occurs in habitats with steppe-like xerophilous vegetation on loamy, loamy-sandy, or loess soils [3]. The species has been reported in natural habitats as well as in semi-natural and anthropogenic habitats such as meadows, pastures, and roadsides [4].

Regarding the food plant of the larvae, *P. thrips* is one of the few European Cossidae and Natura 2000 Lepidoptera species for which the host plant is not known with certainty. According to most studies [1,5,6,7,8,9,10], the larvae feed on *Artemisia* roots. From the observations of [4], it appears that *P. thrips* prefers *Phlomis tuberosa.*

Without knowing its host plant, it is not possible to determine the ecological preferences of the species and thus, determine adequate management. Therefore, knowledge of the host plant and the habitat of the species is critical to its conservation.

*P. thrips* is a western sub-boreal Palaearctic species distributed from eastern Europe to southwestern Siberia [6,11]. In the European part of its distributional range, it has been reported from southern Poland, Hungary [4,12,13,14], Serbia [10], Bulgaria [2,6,10,15,16], Romania [1,7], Ukraine [17] in the central and south of the European part of the Russian Federation [9,18,19,20,21,22,23], Caucasus [11,23,24,25,26,27,28] and from the south of Urals. In Asia it has been reported from Turkey, Iran [29], Transcaucasia in Armenia and Georgia [11,30,31,32,33], in Kazakhstan [34,35], Turkmenistan, in the south of Western Siberia [5,9,22,23,25,36,37,38,39,40,41] (locally in the Omsk Region, Altai Territory, and Khakas). 

The species has been reported in southern Poland (near Przemysl) [42,43], but in the absence of recent reports, the presence of the species remains uncertain. In Hungary, populations are present in the NE (Tisza Valley) and the SE (Hortobágy area) parts of the country, although populations near Budapest are probably extinct [4]. In Serbia, it has been reported from Vidlič, Stara Planina, Suva Planina and Šljivovički Vis [10]. In Bulgaria, it has been reported from Silven, Plovdiv, Burgas, Resselets, Balchik-Topola [21,28,44,45,46,47]. In the Balchik region, it was collected in the early and mid-1990s [48]. It is now extinct in all known areas of Bulgaria, being rediscovered near Sofia in 2020 [10]. From Ukraine, there are reports from the Odesa and Crimea regions, as well as from the northern part of the country near Kyiv [49]. 

**Figure 1:**
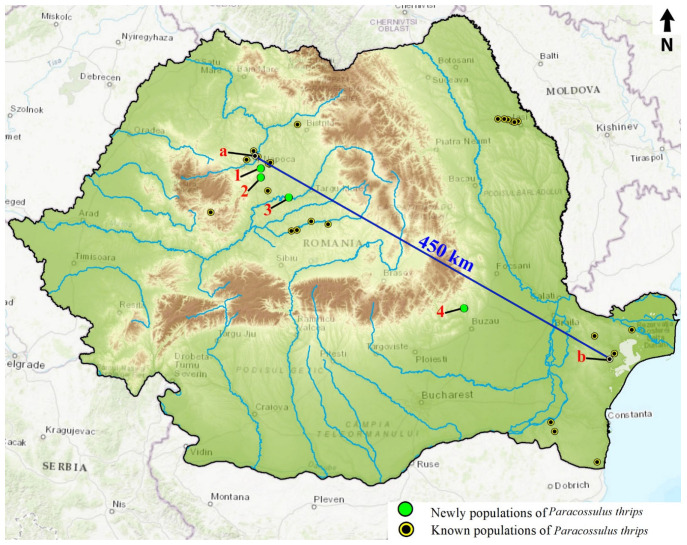
Distribution of Paracossulus thrips populations in Romania. Green dots represent newly populations: **1**—population from Cojocna; **2**—population from Ploscos, **3**—population from Râpa Lechinșa; **4**—population from Vulcanii Noroioși; Black dots represent known population; The two locations for sequences sampling: (**a**)—Jucu de Sus; (**b**)—Babadag, **a** also indicate the studied area.

The first reports of the species on the present territory of Romania date from the beginning of the 20th century, from Transylvania, Szt Gothard [50,51]. More recent reports from Transylvania are from Jucu de Sus [7], Fanatele Clujului (La Copârșaie) [52], Viișoara (Coast of the Moon) [53], Suatu and Căian, Dumbrăveni, La săratura, Rșscruci, Roșia Montană, Sighișoara, Tarnava Mare [7]. From Moldova, it has been reported from Iași [54], Podu Iloaiei [55], Lețcani, Valea Lupului, Valea lui David [56], Valea Ilenei [57,58]. From Dobrogea it has been reported from Visterna [59], Izvoarele, Hagieni-Limanu, Dealurile Beștepe [8,60,61], Urluia, Vedereasa [7] (Figure 1). Nevertheless, its distribution in Romania remains poorly characterized.

In the European part of its range, most populations are found in Romania, followed by the populations in Ukraine [4]. Although *P. thrips* is widespread, the known populations are relatively isolated from one another and no other populational data is known.

The species of interest was described in 1818 by Hübner as *Bombyces thrips*, it is synonymous with *Cossus fuchsianus* Eversmann, 1831; *Cossus kindermanni* Freyer, 1836; *Hypopta trips*, Kjrby, 1892 [9], and was included in the genus *Catopta* Staudinger, 1899 [42,62]. 

The species *thrips* was attributed to the genus *Paracossulus* by [43], considering its placement in the genus *Catopta* to be incorrect. The arguments leading to the placement of *thrips* in the genus *Paracossulus* were morphological aspects such as the rami of the antenna and the structural features of the thorax sclerites [43]. Later, important details were shown in the structure of the male genital apparatus [41].

Morphologically, *P. thrips* is closer to the genera *Parahypopta* and *Cossulus* than *Catopta* [43]. However, a comprehensive phylogenetic study of the *Cossidae* family is needed for a better understanding of the taxonomic placement of the *P. thrips*. The European populations of the *P. thrips* were attributed to ssp. *polonica* [62] which was later synonymized with the nominotypical form [31]. 

In this article, we attempt to fill some of these informational gaps by reporting four new populations in Romania, confirming the host plant for the larvae in the investigated areas, and performing DNA barcoding analyses to establish the taxonomic position of the species from a molecular perspective for the first time.

## 2. Materials and Methods

### 2.1. Field Survey

For observations on the biology and ecology of the species, field studies were conducted, in 2021, in Jucu de Sus, in Natura 2000 ROSCI0295 Dealurile Clujului Est (Cluj County), 26 km from Cluj Napoca, where *P. thrips* has a stable and vigorous population. To identify new populations, we conducted observations in habitats with steppe vegetation in Natura 2000 Protected areas: ROSCI0238 Suatu–Cojocna–Crairât; ROSCI0210 Râpa Lechința and ROSCI0272 Vulcanii Noroioi of Pâclele Mari and Pâclele Mici.

### 2.2. The Identification of the Larval Host Plant

To establish the host plant species with certainty, we used larvae obtained from the eggs of a female captured in Jucu de Sus. We used a sample of 300 eggs/larvae. After hatching, the larvae received fresh roots of *Artemisa absinthium*, *A. maritima, A. vulgaris (Asteraceae)* and tubers and root of *Phlomis tuberosa, Nepeta nuda,* and *Salvia verticillata (Lamiaceae)*. Plants were harvested from steppe habitats where the presence of *P. thrips* is certain. The rhizomes, tubers and roots were cut into slices and placed in the container with the newly emerged larvae so that the larvae could have access to each of the plant species mentioned above.

### 2.3. The Study of Adult Moths

For studying the adult moths, the specimens were lured using light traps. The light source was a fluorescent Hg vapor lamp of 2 × 160 W, a UV blacklight lamp of 2 × 18 W and 3 Led UV light traps 30 LEDs, 5W. The lured specimens were photographed and released. All light traps worked between 20:30 and 02:00. For each studied location, one to five individuals were collected and stored in the Zoological Museum of the Babes-Bolyai University, Cluj Napoca (Table 1 and Table 2). Freshly emerged specimens were collected from Jucu de Sus and kept alive to determine the lifespan and behavior of the adults and to obtain eggs.

### 2.4. Vegetation Analysis

The relevés were realized by using a stratified sampling procedure according to the Braun–Blanquet method. The abundance-dominance notes specific to the phytosociological relevé [63] were replaced by the percentage cover of species, estimated as the horizontal projection of each species on the soil surface. The sampling areas were 25 m^2^ (5 × 5 m). To complete the habitat-level plant species inventory, the relevé method was combined with the linear transect method [64]. The habitats were established according to the literature [65].

The analysis of ecological categories of the investigated phytocoenoses was carried out to indirectly highlight the stationary conditions of each site. Thus, the percentage of species belonging to each ecological category was calculated at the site level. The identified phytotaxa were ecologically characterized according to the literature [66].

### 2.5. Molecular Analysis

#### 2.5.1. Sampling and Collection of Data

Four sequences of *P. thrips* were obtained for this study. The samples were collected from 2 localities (Table 2). Legs were stored in tubes with 96% ethanol. The moths are stored in the Zoological Museum of the Babeș-Bolyai University as vouchers. The sequences were obtained at the Biodiversity Institute of Ontario, Canada. DNA isolation, PCR amplification, and DNA sequencing followed standard protocols [67,68]. 

#### 2.5.2. Sequence Analysis

For comparison with the cladogram of [43], we mined sequences of the Palearctic *Cossidae* from BOLD and NCBI. The full information regarding them is found in Appendix A, Table A1.

Sequence alignment and calculation of genetic distances were done in MEGA X software. Bootstrap analysis (1000 replicates) and the neighbor-joining tree of the COI sequences (the Kimura-two-parameter was used) were also built in MEGA X. We used as the out-group a sequence of *Noctua fimbriata* (Lepidoptera: *Noctuidae*).

## 3. Results

### 3.1. Biology

During the field observations conducted in the Natura 2000 Protected area ROSCI0295 Dealurile Clujului Est (Jucu de Sus), and laboratory survey, we obtained information on adults, eggs, larval behavior, and food plant preferences which represent baseline data on the biology and ecology of the species.

The adult flight period is about four weeks, depending on weather conditions. The flight period may begin from mid-July to mid-late-August. In warm years, the first adults appear as early as late June. Adults are on the wing between 9:30 and 11:30 pm.

**Figure 2:**
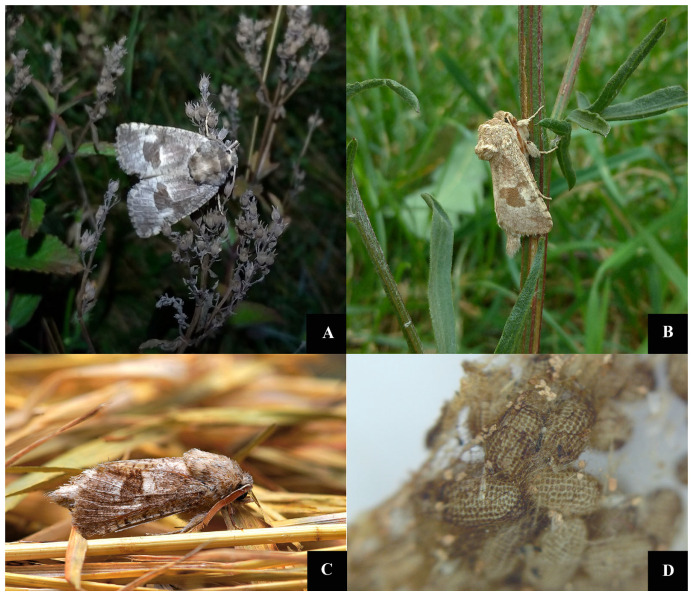
*Paracossulus thrips*—imago (**A**) Female collected from ROSCI0210 Râpa Lechința on 14 August 2021. (**B**) Male collected from ROSCI0238 Suatu–Cojocna–Crairât near Ploscoș (Valea Florilor) on 7 July 2015. (**C**) Male collected from ROSCI0272 Vulcanii Noroioși on 5 August 2015. (**D**) *P. thrips* eggs—photo under stereomicroscope.

According to our observations, adults have low dispersal ability due to heavy flight and short activity/night time. The lifespan of adults held in captivity was 4–5 days; they do not feed (Figure 2A–C). The female lays eggs using the 1.5–2 cm long ovipositor. The eggs are oval, with a larger circumference in the basal third, and are covered with a sticky layer that makes them adhere very well to the substrate (Figure 2D). The available female from Jucu de Sus laid 324 eggs; thereby we assume that a female lays more than 300 eggs.

**Figure 3:**
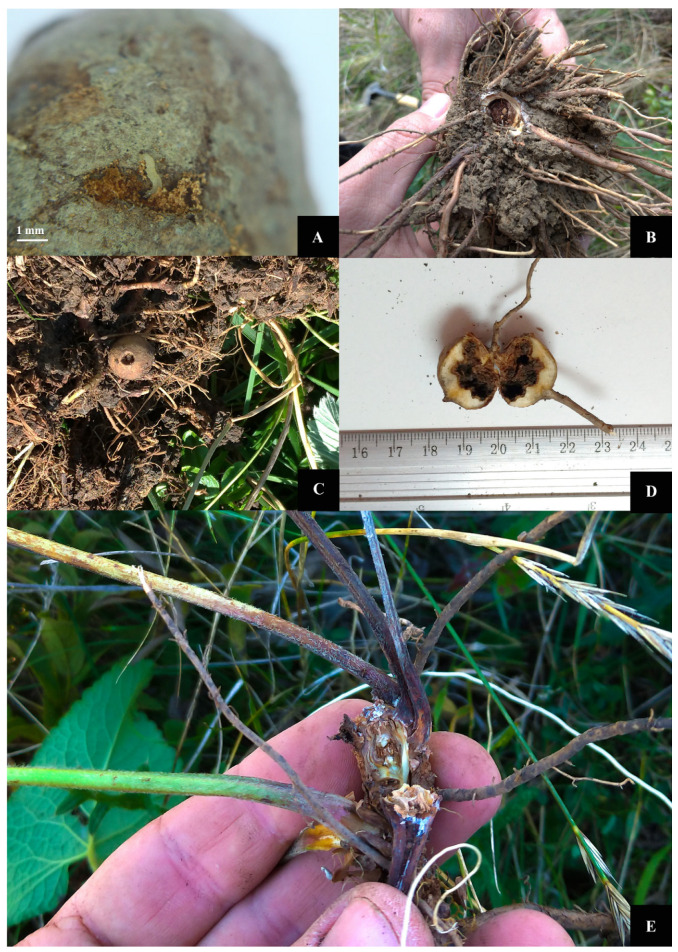
The pattern of damage caused by *Paracossulus thrips* larvae in underground stems and tuberous roots of *Phlomis tuberosa*: (**A**) *P. thrips* larva piercing the tuber of *P. tuberosa;* (**B**) Feeding damage on the rhizome of *P. tuberosa*; (**C**) *P. tuberosa* tuber eaten by *P. thrips* caterpillar; (**D**) *P. tuberosa* tuber showing characteristic *P. thrips* larval damage; (**E**) After hatching, the larvae of *P. thrips* enter the stems of *P. tuberosa* at the base of the leaves that form the basal rosette.

**Figure 4:**
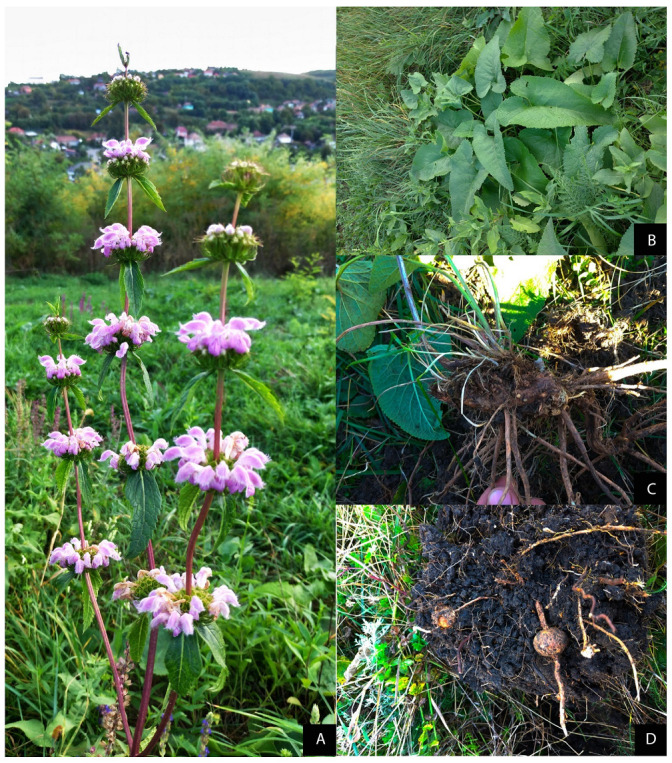
*Phlomis tuberosa*: (**A**) Pink flowers arranged in multiflora verticil; (**B**) triangular lower root leaves; (**C**) Brown rhizome with fibrous roots; (**D**) *P. tuberosa* tubers.

The larvae fed exclusively on the tubers of *Phlomis tuberosa* (Figure 3A–E and Figure 4A–D), although fresh *Artemisia, Nepeta nuda* and *Salvia verticillata* roots were also available. On the field, we found that the newly hatched caterpillars entered the *P. tuberosa* stem in the crown area at the base of the leaves that forms the basal rosette of the plant (Figure 3E).

### 3.2. Vegetation Description

**Figure 5:**
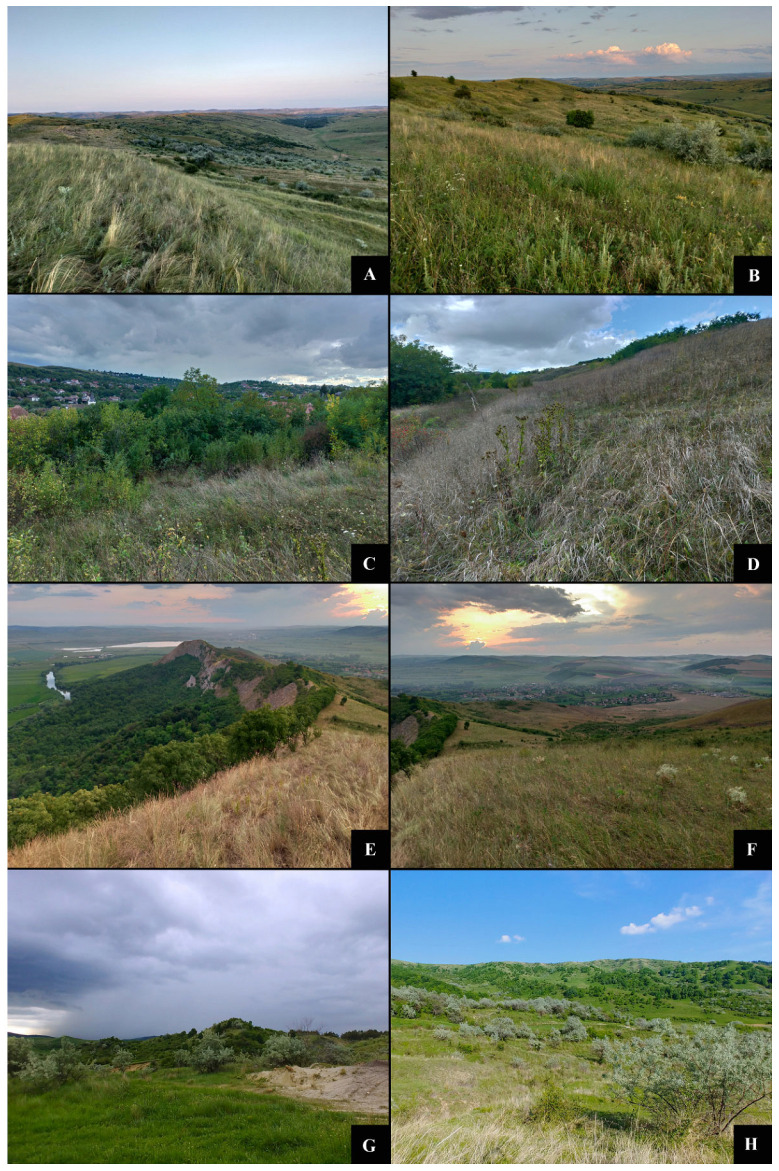
The habitat in sample locations: (**A**,**B**) The Natura 2000 protected area ROSCI0295 Dealurile Clujului Est (Jucu de Sus); (**C**,**D**) The Natura 2000 protected area ROSCI0238 Suatu–Cojocna–Crairât (Cojocna); (**E**,**F**) The Natura 2000 protected site ROSCI0210 Râpa Lechința; (**G**,**H**) The Natura 2000 protected area ROSCI0272 Vulcanii Noroioși from Pâclele Mari and Pâclele Mici.

Natura 2000 protected area ROSCI0295 Dealurile Clujului Est (Jucu de Sus) exhibits a slightly rugged relief, churned up by landslides. The dominant vegetation consists of meadow species interspersed with strips of *Eleagnus angustifolia* planted to stabilize the soil, alongside shrubs of *Prunus spinosa*, *Crataegus monogyna* and *Rosa canina*. *P. tuberosa* grows in the immediate vicinity of shrubs and has a high density, over 1000 stems. The meadow is used as sheep pasture all year round [69] (Figure 5A,B).

**Figure 6:**
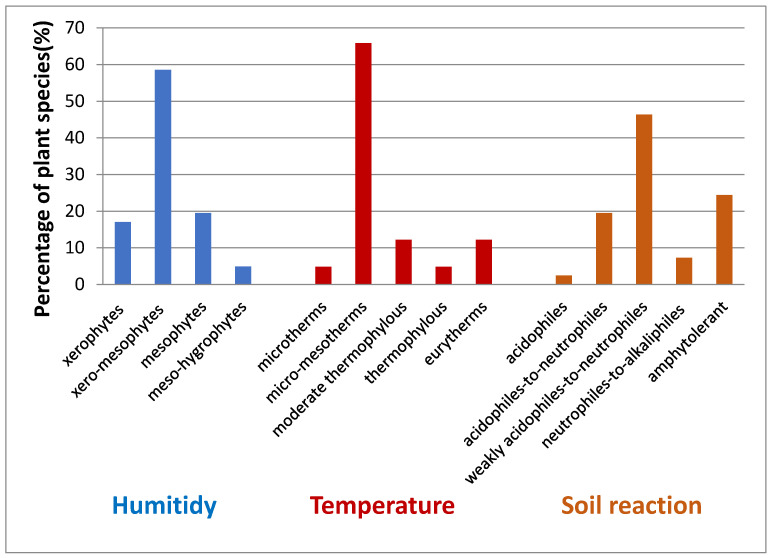
The ecological perspective of investigated vegetation in Natura 2000 ROSCI0295 Dealurile Clujului Est (Jucu de Sus).

Depending on the humidity preferences, dominant species are xero-mesophytes (58.53%), followed by mesophytes (19.51%) and xerophytes (17.07%) species. Regarding the temperature factor, most species are micro-mesotherms (65.85%), followed by moderate thermophilous (12.19%) and eurytherms (12.19%) species and according to the soil reaction, dominant species are weakly acidophiles-to-neutrophiles (46.34%), followed by amphytolerant (24.39%) species (Figure 6).

The Natura 2000 protected area ROSCI0238 Suatu–Cojocna–Crairât consists of a series of fragments of mesoxerophilous and xerophilous steppe meadows arranged on hills with steep slopes, southern aspect, and carbonate soils. Most of these meadows are used as pastures [70] (Figure 5C,D).

In the study area, a part of the meadow area is annually mown and the other part is set on fire. The vegetation structure includes steppe and forest-steppe species, with Stipa lessingiana, Stipa pulcherrima, Stipa tirsa and Stipa capillata dominating. The population of Phlomis tuberosa is located at the base of the slope in an area shaded by a Robinia pseudoacacia plantation and, the meadow is covered by ecotone species characteristic of the former xerothermic forests in the Transylvanian Basin. About 200 stems of P. tuberosa were observed in this area. Analyzing the vegetation, we assume that the surface occupied by *P. tuberosa* was much larger, but was restricted due to the invasion of Robinia pseudoacacia.

**Figure 7:**
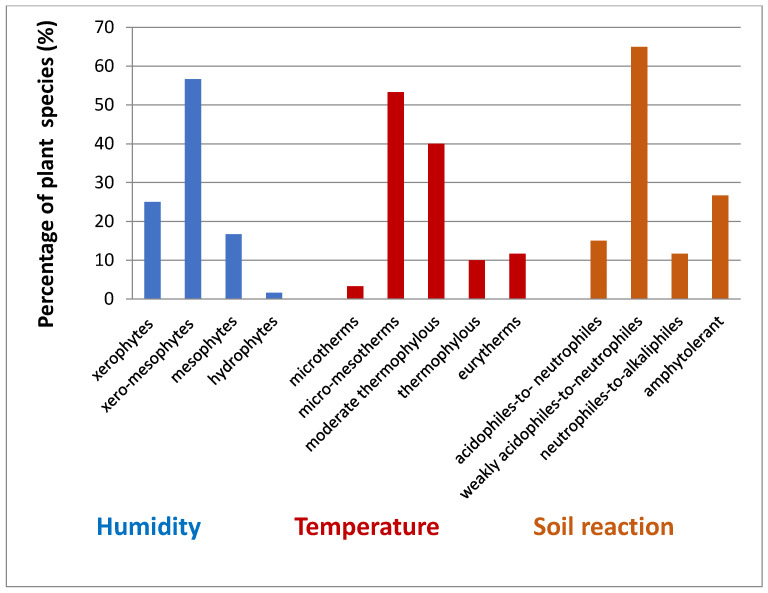
The ecological perspective of investigated vegetation in Natura 2000 ROSCI0238 Suatu–Cojocna–Crairat (Cojocna).

According to the humidity preferences, dominant species are xero-mesophytes (56.66%), followed by xerophytes (25%) and mesophytes (16.66%) species. Regarding the temperature factor, most species are micro-mesotherms (53.33%), followed by moderate thermophilous (40%) species and according to the soil reaction, dominant species are weakly acidophiles-to-neutrophiles (65%), followed by amphytolerant (26.66%) species (Figure 7).

The identified habitats in ROSCI0295 Dealurile Clujului Est (Jucu de Sus) are 6210 Semi-natural dry grasslands and 557 Scrubland facies on calcareous substrates (Festuco-Brometea). In ROSCI0238 Suatu–Cojocna–Crairat–Cojocna, the identified habitat is 6240 * Sub-558 pannonian steppe grasslands. The vegetation structure for both study areas is presented in Appendix A, Table A2.

For the next two sites, we had the existing information in the standard site forms or management plans:

The Natura 2000 protected site ROSCI0210 Râpa Lechința is located on the eastern bank of the river Mureș, near Lechința, and preserves a mosaic of steppe meadows formed on clay-rich and slightly saline soils. The site has an area of 283 hectares and is famous for some rare *Lepidoptera* species such as *Cucullia mixta lorica*, *Hadula dianthi hungarica* and *Conisania poelli ostrogovichi*. The habitat is 6240* Sub-pannonian steppe grasslands [71,72] (Figure 5E,F). 

The Natura 2000 protected area ROSCI0272 Vulcanii Noroioși of Pâclele Mari and Pâclele Mici is located in SE Romania, in the outer part of the Eastern Carpathians [73]. The nature reserve covers an area of 4 ha and is an area of geological and floristic interest, where several protected plant species can be found: *Crambe tataria*, *Iris aphylla ssp. hungarica, Artemisia santonicum, Atriplex tatarica, Ephedra distachya*, etc. The habitat is 1530* Pannonic salt-steppes and salt-marshes [74,75] (Figure 5G,H).

### 3.3. New Populations Identified in Romania

Following the field survey, we discovered four new populations of *P. thrips* in Romania:

- Protected area ROSCI0238 Suatu–Cojocna–Crairât. Near Ploscoș (Valea Florilor) on Dealul Gorgan, four males of P. thrips were observed in 7 July 2015 by Sitar C. and Crișan A.
- Protected area ROSCI0238 Suatu–Cojocna–Crairât. In Cojocna, the larvae were present in the stems of P. tuberosa in the cemetery near the village on 18 September 2021. There is a high density of Phlomis plants at the edge of the cemetery. (Iacob G., Sitar C., Beldean M.) (Figure 5C,D).
- Protected area ROSCI0210 Râpa Lechința, near Lechința (Mureș County). On 14 August 2021, a light trap deployed by Iacob G. and Sitar C attracted a female P. thrips (Figure 5E,F).
- Protected area ROSCI0272 Vulcanii Noroioși of Pâclele Mari and Pâclele Mici. On 5 August 2015 a light trap deployed by Aurelian V.M. attracted a male P. thrips (Figure 5G,H).

### 3.4. Sequence Analysis

The four sequences of *P. thrips* were submitted to GenBank; they are the first published sequences of this species (accession numbers OK314991, OK314992, OK314993, OK314994). The sequences belong to individuals from two populations: three from Jucu de Sus (Cluj County) and one from Pădurea Babadag (Tulcea County) (Table 2). The two populations are 450 km apart and are geographically isolated by a natural barrier (Carpathian mountains) (Figure 1). The genetic distance between individuals is 0 and 0.002 (Table 3). The values of genetic distance between the sequences of *P. thrips* and the out-group are 0.6857 and 0.6846, respectively (Table 3). Full sequences of *P. thrips* are available in the Appendix A, Table A3.

**Figure 8:**
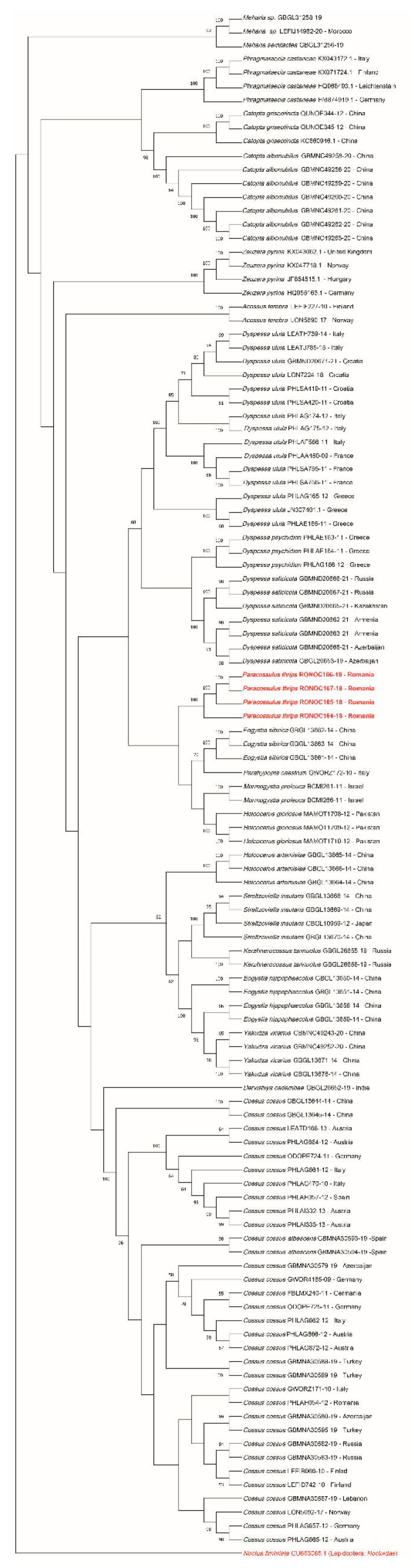
Neighbor-joining (NJ) tree of COI barcodes of Palearctic *Cossidae.* The tree includes sequences of *P. thrips* and as out-group *Noctua fimbriata* (Lepidoptera: *Noctuidae*), all highlighted in red.

**Figure 9:**
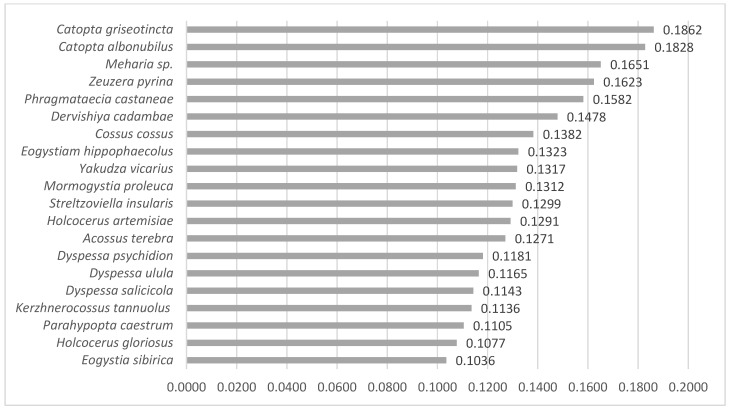
The average values of the genetic distances of the analyzed species towards the *P. thrips* species.

In the molecular analysis, we examined 21 Palearctic species of *Cossidae*. Our dataset consisted of 191 sequences mined from BOLD and NCBI (see the electronic Supplementary Materials, Annex S1). The neighbor-joining (NJ) tree was made based on 113 of the sequences (Figure 8) for comparison with the cladogram of [43]. The lowest values of the recorded genetic distances are 0.0041 and 0.0166 between species *Yakudza vicarius* and *Eogystia hippophaecolus*, respectively, 0.0622 between *Streltzoviella insularis* and *Kerzhnerocossus tannuolus* (see the electronic Supplementary Materials, Annex S2). The nearest species to *P. thrips* is *Eogystia sibirica* (0.1036) (Figure 9). The matrix with the genetic distances between *P. thrips* and the species from the same clade is presented in Table 3. *Catopta griseotincta* is the most distant species from *P. thrips* (0.1862), after the out-group (Figure 9). All genetic distances between specimens of *P. trips* and specimens of the genus *Catopta* are presented in (Table 4). The average genetic distance of the entire analyzed group is 0.1231 (without out-group) and 0.1328 (with out-group). Genetic distances for all sequences are available in the electronic Supplementary Material, Annex S2.

Our results show that DNA barcoding worked well in discriminating *P. thrips* sequences. Our results also show that DNA barcoding worked well in discriminating the 21 species considered in the analysis.

## 4. Discussion

*P. thrips* is susceptible to extinction as a result of the geographical isolation of extant populations. There is also a lack of information on larval host plants, types of habitats, or population densities and dynamics. As such, this species is given protected status in Europe. In the first part of the study, we attempted to establish with certainty the larval host plant. To date, most authors consider *Artemisia* sp. to be the larval host plant [1,5,6,7,8,9,10]. Ref. [4] states that the host plant is *P. tuberosa*, but there are no published studies to confirm this so far. Our studies confirm that *P. tuberosa* is the larval host plant for *P. thrips* in Romania

The limited mobility and short flight time of adults also limit the colonization of new habitats. Thus, any pressure exerted on the remaining habitat by external factors will also affect the populations of *P. thrips.* For this reason, we consider the knowledge of the ecological preferences of *P. tuberosa* and population dynamics to be extremely important in order to better formulate any future management and protection implications.

*Phlomis tuberosa* is a continental Eurasian species. In Romania, it has been reported mainly in stands with woody vegetation, such as forests, bushes, vineyards, but also in hay meadows. It is often found in forest-steppe regions [76]. It occurs in stands with slight or no human impact, being considered oligohemerobic to mesohemerobic [77]. From an ecological point of view, the species is associated with xerothermic oak and deciduous forests, which are conditioned azonally by the orographic and edaphic particularities (*Quercetalia pubescentis* Br.–Bl 1931 em.), as well as with xeric grassland communities, established on cleared slopes in the oak area [66].

Thorough knowledge of habitat preferences can provide the data needed to identify potential habitats, including the identification of any new populations of *P. thrips*. Although most of the moth populations in the European part of the range are on the territory of Romania, the distribution of the species in this country is not yet fully characterized. For example, the presence of the nearby Hungarian populations from Hortobágy and Gyula [4] possibly indicates the existence of a population of *P. thrips* in the western part of Romania.

Although the light traps worked for almost 6 h, between 20:30 and 02:00, all individuals were observed between 09:30 and 10:30. The short flight interval, the isolated populations, and the direct observations made on the field indicate a low dispersal capacity of the individuals. Another limiting factor that contributes to the reduced dispersion is the short life span of adults. This aspect is also characteristic of other species of *Cossidae* [78] due to the atrophied buccal apparatus of adults that do not feed. 

The second part of our study attempted to confirm the current taxonomic placement of *P. thrips*. Until recently, *P. thrips* was classified as *Catopta* [62], but in 1990 the species was included by [43] in the distinct genus *Paracosulus*. Ref. [43] presents a cladogram (based on the external morphology of the adults) that places the species *P. thrips* closer to the genera *Dyspessacossus*, *Parahypopta*, and *Dyspessa*. Furthermore, the genus *Catopta* is assigned to the subfamily *Catoptinae* [79] that was described based on the apomorphic features of the genitals of both sexes [79].

This framing is also supported by our DNA barcoding analysis. The sequences of *P. thrips* are grouped in the same cluster and form a clade with the species *Eogystia sibirica, Parahypopta caestrum, Mormogystia proleuca,* and *Holcocerus gloriosus.* All of them are grouped into a larger clade with the genus *Dyspessa* represented by three species: *D. ulula, D. Psychidion,* and *D. salicicola.* Thus our preliminary genetic data confirms the morphological studies of [43] and they argue the use of the genus *Paracossulus* instead of the genus *Catopta.* This is supported both by the position of the two genera in the phylogenetic tree and by the genetic distance between *Paracossulus* and *Catopta* which is on average 0.1832 (Table 4). A comparison of *P. thrips* with species of the genera *Dyspesacossus* and *Cossulus* was not possible due to the lack of available sequences.

Despite the geographical isolation, there are no significant differences between the sequences of *P. thrips* from the specimens from Jucu de Sus and the specimen from Babadag Forest (Figure 1) (Table 3). This lack of genetic diversity between the various isolated populations requires further studies with different methods.

*P. thrips* and its habitat are directly threatened by overgrazing (especially by sheep and/or goats) at most or all sites, by mowing of meadows or by other land mismanagement practices which lead to rapid habitat degradation. As such, conservation measures for *P. thrips* are acutely needed to prevent these threats and management practices must be substantiated based on available data. There was a fine mosaic of over-exploited to abandoned places. All meadows as habitat were grazed and mown for hay and part of the steppe-like meadows were commonly burned. Habitat changes similar to those happening in the rest of Europe are present here too. Namely, meadows are changing to more homogenous ones, without the fine mosaic of micro-habitats. It seems that for a species such as *P. thrips* traditional burning (in spring, with cold nights) is not problematic. Food plants are robust enough to protect larvae inside of the roots and burning provides free space for new seedlings of *P. tuberosa*. Otherwise without burning the populations survive only on old plants of *P. tuberosa* and it should be very problematic in the future. 

Studies on the dispersal capacity of the species and estimates of populations are needed in the nearest future.

## Figures and Tables

**Table 1:** Voucher and specimens’ details from studied locations.

Species	Voucher	Locality	Natura 2000 Site	County	Sex	Age
*Paracossulus thrips*	LEP007309	Jucu de Sus	ROSCI0295	Cluj	female	imago
	LEP007310	Jucu de Sus	ROSCI0295	Cluj	male	imago
	LEP007311	Cojocna	ROSCI0238	Cluj	-	larva
	LEP007312	Râpa Lechința, Iernut	ROSCI0238	Mureș	female	imago
	LEP007313	Ploscoș, Gorgan Hill	ROSCI0210	Cluj	male	imago
	LEP007314	Vulcanii Noroioși	ROSCI0272	Buzău	male	imago

**Table 2:** The details of specimens used for DNA barcoding.

Sequence_ID	Species	Collected By	Collection Date	Country	County	Locality
RONOC164-18	*Paracossulus thrips*	Sitar C.	21 July 2016	Romania	Cluj	Jucu de Sus
RONOC165-18	*Paracossulus thrips*	Sitar C.	5 August 2016	Romania	Cluj	Jucu de Sus
RONOC166-18	*Paracossulus thrips*	Sitar C.	21 July 2016	Romania	Cluj	Jucu de Sus
RONOC167-18	*Paracossulus thrips*	Szekely L.	26 August 2011	Romania	Tulcea	Babadag Forest

**Table 3:** The genetic distances between *P. thrips* and the species from the same clade. The genetic distances from the out-group are presented in column 14.

SEQUENCE_ID	SPECIES		1	2	3	4	5	6	7	8	9	10	11	12	13	14
RONOC164-18	*Paracossulus thrips*	1		0.0021	0.0021	0.0021	0.1037	0.1058	0.1058	0.1120	0.1328	0.1328	0.1120	0.1079	0.1079	0.6846
RONOC165-18	*Paracossulus thrips*	2			0.0000	0.0000	0.1017	0.1037	0.1037	0.1100	0.1307	0.1307	0.1100	0.1058	0.1058	0.6867
RONOC166-18	*Paracossulus thrips*	3				0.0000	0.1017	0.1037	0.1037	0.1100	0.1307	0.1307	0.1100	0.1058	0.1058	0.6867
RONOC167-18	*Paracossulus thrips*	4					0.1017	0.1037	0.1037	0.1100	0.1307	0.1307	0.1100	0.1058	0.1058	0.6867
GBGL13661-14	*Eogystia sibirica*	5						0.0021	0.0021	0.0705	0.1058	0.1058	0.0954	0.0954	0.0954	0.6888
GBGL13662-14	*Eogystia sibirica*	6							0.0000	0.0726	0.1079	0.1079	0.0975	0.0975	0.0975	0.6909
GBGL13663-14	*Eogystia sibirica*	7								0.0726	0.1079	0.1079	0.0975	0.0975	0.0975	0.6909
GWORZ172-10	*Parahypopta caestrum*	8									0.1183	0.1183	0.1037	0.0996	0.0996	0.6846
BCMI261-11	*Mormogystia proleuca*	9										0.0000	0.1100	0.1100	0.1100	0.6805
BCMI266-11	*Mormogystia proleuca*	10											0.1100	0.1100	0.1100	0.6805
MAMOT1708-12	*Holcocerus gloriosus*	11												0.0041	0.0041	0.6888
MAMOT1709-12	*Holcocerus gloriosus*	12													0.0000	0.6929
MAMOT1710-12	*Holcocerus gloriosus*	13														0.6929
GU663065.1	*Noctua fimbriata*	14														

**Table 4:** The matrix with the genetic distances between *P. thrips* and *Catopta* species.

SEQUENCE_ID	SPECIES		1	2	3	4	5	6	7	8	9	10	11	12	13
RONOC164-18	*Paracossulus thrips*	1		0.0021	0.0021	0.0021	0.1846	0.1846	0.1846	0.1826	0.1846	0.1846	0.1846	0.1846	0.1846
RONOC165-18	*Paracossulus thrips*	2			0.0000	0.0000	0.1867	0.1867	0.1826	0.1805	0.1826	0.1826	0.1826	0.1826	0.1826
RONOC166-18	*Paracossulus thrips*	3				0.0000	0.1867	0.1867	0.1826	0.1805	0.1826	0.1826	0.1826	0.1826	0.1826
RONOC167-18	*Paracossulus thrips*	4					0.1867	0.1867	0.1826	0.1805	0.1826	0.1826	0.1826	0.1826	0.1826
KC860946.1	*Catopta griseotincta*	5						0.0000	0.1079	0.1100	0.1079	0.1079	0.1079	0.1079	0.1079
QUNOE344-12	*Catopta griseotincta*	6							0.1079	0.1100	0.1079	0.1079	0.1079	0.1079	0.1079
GBMNC49256-20	*Catopta albonubilus*	7								0.0021	0.0000	0.0000	0.0000	0.0000	0.0000
GBMNC49258-20	*Catopta albonubilus*	8									0.0021	0.0021	0.0021	0.0021	0.0021
GBMNC49259-20	*Catopta albonubilus*	9										0.0000	0.0000	0.0000	0.0000
GBMNC49260-20	*Catopta albonubilus*	10											0.0000	0.0000	0.0000
GBMNC49261-20	*Catopta albonubilus*	11												0.0000	0.0000
GBMNC49262-20	*Catopta albonubilus*	12													0.0000
GBMNC49263-20	*Catopta albonubilus*	13													

## Data Availability

Not applicable.

## Supplementary Materials

The following are available online at https://www.mdpi.com/article/10.3390/insects12121087/s1, Annex S1: All sequences mined from BOLD & NCBI; Annex S2: MEGA- Genetic distances.

## Appendix A

**Table A1:** The full information for the sequences mined from GenBank.

BOLD_ID	NCBI_ID	Species	Country	Reference Title	Authors	Collection Date
LON5690-17	\	*Acossus terebra*	Norway	iBOL Data Release	iBOL	20 July 2010
LEFIF227-10	HM874920	*Acossus terebra*	Finland	Species-Level Para- and Polyphyly in DNA Barcode Gene Trees: Strong Operational Bias in European Lepidoptera.	Mutanen, M. et al., 2016	\
GBMNC49256-20	MF052579	*Catopta albonubilus*	China	Using full-length metabarcoding and DNA barcoding to infer community assembly for speciose taxonomic groups: a case study.	Hao, M.-D. et al., 2020	\
GBMNC49263-20	MF051783	*Catopta albonubilus*	China	Using full-length metabarcoding and DNA barcoding to infer community assembly for speciose taxonomic groups: a case study.	Hao, M.-D. et al., 2020	\
GBMNC49258-20	MF051921	*Catopta albonubilus*	China	Using full-length metabarcoding and DNA barcoding to infer community assembly for speciose taxonomic groups: a case study.	Hao, M.-D. et al., 2020	\
GBMNC49259-20	MF051897	*Catopta albonubilus*	China	Using full-length metabarcoding and DNA barcoding to infer community assembly for speciose taxonomic groups: a case study.	Hao, M.-D. et al., 2020	\
GBMNC49260-20	MF051847	*Catopta albonubilus*	China	Using full-length metabarcoding and DNA barcoding to infer community assembly for speciose taxonomic groups: a case study.	Hao, M.-D. et al., 2020	\
GBMNC49261-20	MF051833	*Catopta albonubilus*	China	Using full-length metabarcoding and DNA barcoding to infer community assembly for speciose taxonomic groups: a case study.	Hao, M.-D. et al., 2020	\
GBMNC49262-20	MF051788	*Catopta albonubilus*	China	Using full-length metabarcoding and DNA barcoding to infer community assembly for speciose taxonomic groups: a case study.	Hao, M.-D. et al., 2020	\
QUNOE344-12	KC860946	*Catopta griseotincta*	China	A brief review of genus Catopta Staudinger, 1899 (Lepidoptera: *Cossidae*) with description of a new species from China	Yakovlev, R.V. et al., 2013	30 July 2011
QUNOE345-12	KC860945	*Catopta griseotincta*	China	A brief review of genus Catopta Staudinger, 1899 (Lepidoptera: *Cossidae*) with description of a new species from China	Yakovlev, R.V. et al., 2013	30 July 2011
KC860946	QUNOE344	*Catopta griseotincta*	China	A brief review of genus Catopta Staudinger, 1899 (Lepidoptera: *Cossidae*) with description of a new species from China	Yakovlev, R.V. et al., 2013	30 July 2011
FBLMX240-11	KX040722	*Cossus cossus*	Germany	iBOL Data Release	iBOL	14 July 2010
GWORZ171-10	HM914074	*Cossus cossus*	Italy	iBOL Data Release	iBOL	20 July 1996
LON5692-17	\	*Cossus cossus*	Norway	iBOL Data Release	iBOL	15 June 2013
PHLAH054-12	KX045460	*Cossus cossus*	Romania	Species-Level Para- and Polyphyly in DNA Barcode Gene Trees: Strong Operational Bias in European Lepidoptera.	Mutanen, M. et al., 2016	26 July 1999
PHLAH357-12	\	*Cossus cossus*	Spain	Species-Level Para- and Polyphyly in DNA Barcode Gene Trees: Strong Operational Bias in European Lepidoptera.	Mutanen, M. et al., 2016	1 September 2005
GBMNA30579-19	MK440671	*Cossus cossus*	Azerbaijan	A DNA-based description of a new carpenter moth species (Lepidoptera: *Cossidae*) from Morocco	Yakovlev, R.V. et al., 2019	1 July 2008
GBMNA30580-19	MK440678	*Cossus cossus*	Azerbaijan	A DNA-based description of a new carpenter moth species (Lepidoptera: *Cossidae*) from Morocco	Yakovlev, R.V. et al., 2019	1 July 2008
GBMNA30582-19	MK440673	*Cossus cossus*	Russia	A DNA-based description of a new carpenter moth species (Lepidoptera: *Cossidae*) from Morocco	Yakovlev, R.V. et al., 2019	3 June 2006
GBMNA30583-19	MK440674	*Cossus cossus*	Russia	A DNA-based description of a new carpenter moth species (Lepidoptera: *Cossidae*) from Morocco	Yakovlev, R.V. et al., 2019	3 June 2006
GBMNA30587-19	MK440667	*Cossus cossus*	Lebanon	A DNA-based description of a new carpenter moth species (Lepidoptera: *Cossidae*) from Morocco	Yakovlev, R.V. et al., 2019	1 May 2006
GBMNA30588-19	MK440668	*Cossus cossus*	Turkey	A DNA-based description of a new carpenter moth species (Lepidoptera: *Cossidae*) from Morocco	Yakovlev, R.V. et al., 2019	1 June 2002
GBMNA30589-19	MK440669	*Cossus cossus*	Turkey	A DNA-based description of a new carpenter moth species (Lepidoptera: *Cossidae*) from Morocco	Yakovlev, R.V. et al., 2019	1 June 2002
GBMNA30595-19	MK440679	*Cossus cossus*	Turkey	A DNA-based description of a new carpenter moth species (Lepidoptera: *Cossidae*) from Morocco	Yakovlev, R.V. et al., 2019	3 August 2005
LEATD168-13	\	*Cossus cossus*	Austria	DNA barcode library for Lepidoptera from South Tyrol and Tyrol (Italy, Austria)—Impetus for integrative species discrimination in the 21st Century	Huemer, P. & Heber, P.D.N., 2016	7 June 2013
PHLAI332-13	\	*Cossus cossus*	Austria	DNA barcode library for Lepidoptera from South Tyrol and Tyrol (Italy, Austria)—Impetus for integrative species discrimination in the 21st Century	Huemer, P. & Heber, P.D.N., 2016	2 June 2012
PHLAI333-13	\	*Cossus cossus*	Austria	DNA barcode library for Lepidoptera from South Tyrol and Tyrol (Italy, Austria)—Impetus for integrative species discrimination in the 21st Century	Huemer, P. & Heber, P.D.N., 2016	19 June 2012
PHLAC470-10	JF860045	*Cossus cossus*	Italy	DNA-barcoding von schmetterlingen (Lepidoptera) in Waldstandorten Südtirols (IT01 ritten und IT02 montiggl)	Huemer, P. & Hebert, P.D.N., 2012	4 June 2010
GBGL13644-14	KC791441	*Cossus cossus*	China	Forest Resource Conservation Department, Key Laboratory for Silviculture and Conservation of Ministry of Education	Li, J. & Chen, M.	10 August 2012
GBGL13645-14	KC791442	*Cossus cossus*	China	Forest Resource Conservation Department, Key Laboratory for Silviculture and Conservation of Ministry of Education	Li, J. & Chen, M.	10 August 2012
GWOR4165-09	KX070766	*Cossus cossus*	Germany	Species-Level Para- and Polyphyly in DNA Barcode Gene Trees: Strong Operational Bias in European Lepidoptera.	Mutanen, M. et al., 2016	23 June 2008
ODOPE724-11	KX040920	*Cossus cossus*	Germany	Species-Level Para- and Polyphyly in DNA Barcode Gene Trees: Strong Operational Bias in European Lepidoptera.	Mutanen, M. et al., 2016	17 June 2005
ODOPE725-11	KX040085	*Cossus cossus*	Germany	Species-Level Para- and Polyphyly in DNA Barcode Gene Trees: Strong Operational Bias in European Lepidoptera.	Mutanen, M. et al., 2016	28 June 2008
PHLAG857-12	\	*Cossus cossus*	Germany	Species-Level Para- and Polyphyly in DNA Barcode Gene Trees: Strong Operational Bias in European Lepidoptera.	Mutanen, M. et al., 2016	7 June 1991
PHLAG861-12	\	*Cossus cossus*	Italy	Species-Level Para- and Polyphyly in DNA Barcode Gene Trees: Strong Operational Bias in European Lepidoptera.	Mutanen, M. et al., 2016	22 August 1993
PHLAG862-12	\	*Cossus cossus*	Italy	Species-Level Para- and Polyphyly in DNA Barcode Gene Trees: Strong Operational Bias in European Lepidoptera.	Mutanen, M. et al., 2016	10 July 1994
PHLAG864-12	\	*Cossus cossus*	Austria	Species-Level Para- and Polyphyly in DNA Barcode Gene Trees: Strong Operational Bias in European Lepidoptera.	Mutanen, M. et al., 2016	24 June 1994
PHLAG865-12	\	*Cossus cossus*	Austria	Species-Level Para- and Polyphyly in DNA Barcode Gene Trees: Strong Operational Bias in European Lepidoptera.	Mutanen, M. et al., 2016	14 March 2002
PHLAG866-12	\	*Cossus cossus*	Austria	Species-Level Para- and Polyphyly in DNA Barcode Gene Trees: Strong Operational Bias in European Lepidoptera.	Mutanen, M. et al., 2016	5 July 1989
LEFIB066-10	HM870975	*Cossus cossus*	Finlad	Testing DNA Barcode Performance in 1000 Species of European Lepidoptera: Large Geographic Distances Have Small Genetic Impacts	Huemer, P. et al., 2014	/
PHLAG872-12	KM572562	*Cossus cossus*	Austria	Testing DNA Barcode Performance in 1000 Species of European Lepidoptera: Large Geographic Distances Have Small Genetic Impacts	Huemer, P. et al., 2014	28 June 2011
LEFID742-10	HM873499	*Cossus cossus*	Finland	Testing DNA Barcode Performance in 1000 Species of European Lepidoptera: Large Geographic Distances Have Small Genetic Impacts	Huemer, P. et al., 2014	14 July 2007
GBMNA30593-19	MK440665	*Cossus cossus albescens*	Spain	A DNA-based description of a new carpenter moth species (Lepidoptera: *Cossidae*) from Morocco	Yakovlev, R.V. et al., 2019	29 June 1990
GBMNA30594-19	MK440666	*Cossus cossus albescens*	Spain	A DNA-based description of a new carpenter moth species (Lepidoptera: *Cossidae*) from Morocco	Yakovlev, R.V. et al., 2019	16 June 2007
GBGL26652-19	MG279391	*Dervishiya cadambae*	India	First report of occurrence of *Dervishiya cadambae* (Moore, 1865) on grapes, *Vitis vinifera* L. in India Unpublished	Yadav, D.S. et al.	\
PHLAE163-11	JN307399	*Dyspessa psychidion*	Greece	Species-Level Para- and Polyphyly in DNA Barcode Gene Trees: Strong Operational Bias in European Lepidoptera.	Mutanen, M. et al., 2016	19 May 2009
PHLAE164-11	JN307400	*Dyspessa psychidion*	Greece	Species-Level Para- and Polyphyly in DNA Barcode Gene Trees: Strong Operational Bias in European Lepidoptera.	Mutanen, M. et al., 2016	19 May 2009
PHLAG166-12	\	*Dyspessa psychidion*	Greece	Species-Level Para- and Polyphyly in DNA Barcode Gene Trees: Strong Operational Bias in European Lepidoptera.	Mutanen, M. et al., 2016	16 May 2009
GBMND20662-21	MW170375	*Dyspessa salicicola*	Armenia	A new species of Carpenter-Moths (Lepidoptera, *Cossidae*) from Tarbagatai (NE Kazakhstan) and Altai (SW Siberia, Russia) Mountains	Yakovlev, R.V. et al., 2020	\
GBMND20663-21	MW170376	*Dyspessa salicicola*	Armenia	A new species of Carpenter-Moths (Lepidoptera, *Cossidae*) from Tarbagatai (NE Kazakhstan) and Altai (SW Siberia, Russia) Mountains	Yakovlev, R.V. et al., 2020	\
GBMND20665-21	MW170379	*Dyspessa salicicola*	Kazakhstan	A new species of Carpenter-Moths (Lepidoptera, *Cossidae*) from Tarbagatai (NE Kazakhstan) and Altai (SW Siberia, Russia) Mountains	Yakovlev, R.V. et al., 2020	\
GBMND20666-21	MW170380	*Dyspessa salicicola*	Russia	A new species of Carpenter-Moths (Lepidoptera, *Cossidae*) from Tarbagatai (NE Kazakhstan) and Altai (SW Siberia, Russia) Mountains	Yakovlev, R.V. et al., 2020	\
GBMND20667-21	MW170381	*Dyspessa salicicola*	Russia	A new species of Carpenter-Moths (Lepidoptera, *Cossidae*) from Tarbagatai (NE Kazakhstan) and Altai (SW Siberia, Russia) Mountains	Yakovlev, R.V. et al., 2020	\
GBMND20668-21	MW170377	*Dyspessa salicicola*	Azerbaijan	A new species of Carpenter-Moths (Lepidoptera, *Cossidae*) from Tarbagatai (NE Kazakhstan) and Altai (SW Siberia, Russia) Mountains	Yakovlev, R.V. et al., 2020	\
GBGL26653-19	MF596152	*Dyspessa salicicola*	Azerbaijan	The taxonomic status of *Cossus cossus afghanistanus* (Lepidoptera, *Cossidae*) from Afghanistan: insights from molecular and morphological data.	Shapoval, N.A. et al., 2017	1 July 2008
LON7224-18	\	*Dyspessa ulula*	Croatia	\	\	1 June 2018
GBMND20671-21	MW170384	*Dyspessa ulula*	Croatia	A new species of Carpenter-Moths (Lepidoptera, *Cossidae*) from Tarbagatai (NE Kazakhstan) and Altai (SW Siberia, Russia) Mountains	Yakovlev, R.V. et al., 2020	\
LEATH739-14	\	*Dyspessa ulula*	Italy	DNA barcode library for Lepidoptera from South Tyrol and Tyrol (Italy, Austria)–Impetus for integrative species discrimination in the 21st Century	Huemer, P. & Heber, P.D.N., 2016	6 June 2014
LEATJ785-15	\	*Dyspessa ulula*	Italy	DNA barcode library for Lepidoptera from South Tyrol and Tyrol (Italy, Austria)–Impetus for integrative species discrimination in the 21st Century	Huemer, P. & Heber, P.D.N., 2016	18 June 2015
PHLAA450-09	HM425963	*Dyspessa ulula*	France	Species-Level Para- and Polyphyly in DNA Barcode Gene Trees: Strong Operational Bias in European Lepidoptera.	Mutanen, M. et al., 2016	6 June 2009
PHLAE165-11	JN307401	*Dyspessa ulula*	Greece	Species-Level Para- and Polyphyly in DNA Barcode Gene Trees: Strong Operational Bias in European Lepidoptera.	Mutanen, M. et al., 2016	17 May 2009
PHLAF586-11	\	*Dyspessa ulula*	Italy	Species-Level Para- and Polyphyly in DNA Barcode Gene Trees: Strong Operational Bias in European Lepidoptera.	Mutanen, M. et al., 2016	11 June 2009
PHLAG165-12	\	*Dyspessa ulula*	Greece	Species-Level Para- and Polyphyly in DNA Barcode Gene Trees: Strong Operational Bias in European Lepidoptera.	Mutanen, M. et al., 2016	15 July 2009
PHLAG174-12	\	*Dyspessa ulula*	Italy	Species-Level Para- and Polyphyly in DNA Barcode Gene Trees: Strong Operational Bias in European Lepidoptera.	Mutanen, M. et al., 2016	16 July 2010
PHLAG175-12	\	*Dyspessa ulula*	Italy	Species-Level Para- and Polyphyly in DNA Barcode Gene Trees: Strong Operational Bias in European Lepidoptera.	Mutanen, M. et al., 2016	16 July 2010
PHLSA419-11	\	*Dyspessa ulula*	Croatia	Species-Level Para- and Polyphyly in DNA Barcode Gene Trees: Strong Operational Bias in European Lepidoptera.	Mutanen, M. et al., 2016	25 May 1995
PHLSA420-11	\	*Dyspessa ulula*	Croatia	Species-Level Para- and Polyphyly in DNA Barcode Gene Trees: Strong Operational Bias in European Lepidoptera.	Mutanen, M. et al., 2016	25 May 1995
PHLSA755-11	\	*Dyspessa ulula*	France	Species-Level Para- and Polyphyly in DNA Barcode Gene Trees: Strong Operational Bias in European Lepidoptera.	Mutanen, M. et al., 2016	20 May 2010
PHLSA756-11	\	*Dyspessa ulula*	France	Species-Level Para- and Polyphyly in DNA Barcode Gene Trees: Strong Operational Bias in European Lepidoptera.	Mutanen, M. et al., 2016	20 May 2010
GBGL13650-14	KC791450	*Eogystia hippophaecolus*	China	Forest Resource Conservation Department, Key Laboratory for Silviculture and Conservation of Ministry of Education	Li, J. & Chen, M.	10 August 2012
GBGL13651-14	KC791451	*Eogystia hippophaecolus*	China	Forest Resource Conservation Department, Key Laboratory for Silviculture and Conservation of Ministry of Education	Li, J. & Chen, M.	10 August 2012
GBGL13658-14	KC791458	*Eogystia hippophaecolus*	China	Forest Resource Conservation Department, Key Laboratory for Silviculture and Conservation of Ministry of Education	Li, J. & Chen, M.	28 July 2012
GBGL13659-14	KC791459	*Eogystia hippophaecolus*	China	Forest Resource Conservation Department, Key Laboratory for Silviculture and Conservation of Ministry of Education	Li, J. & Chen, M.	28 July 2012
GBGL13661-14	KC791482	*Eogystia sibirica*	China	Forest Resource Conservation Department, Key Laboratory for Silviculture and Conservation of Ministry of Education	Li, J. & Chen, M.	28 July 2012
GBGL13662-14	KC791483	*Eogystia sibirica*	China	Forest Resource Conservation Department, Key Laboratory for Silviculture and Conservation of Ministry of Education	Li, J. & Chen, M.	28 July 2012
GBGL13663-14	KC791484	*Eogystia sibirica*	China	Forest Resource Conservation Department, Key Laboratory for Silviculture and Conservation of Ministry of Education	Li, J. & Chen, M.	28 July 2012
GBGL13664-14	KC791443	*Holcocerus artemisiae*	China	Forest Resource Conservation Department, Key Laboratory for Silviculture and Conservation of Ministry of Education	Li, J. & Chen, M.	8 August 2012
GBGL13665-14	KC791444	*Holcocerus artemisiae*	China	Forest Resource Conservation Department, Key Laboratory for Silviculture and Conservation of Ministry of Education	Li, J. & Chen, M.	8 August 2012
GBGL13666-14	KC791445	*Holcocerus artemisiae*	China	Forest Resource Conservation Department, Key Laboratory for Silviculture and Conservation of Ministry of Education	Li, J. & Chen, M.	8 August 2012
MAMOT1708-12	KX860954	*Holcocerus gloriosus*	Pakistan	Mapping global biodiversity connections with DNA barcodes: Lepidoptera of Pakistan	Muhammad A. et al., 2017	9 May 2012
MAMOT1709-12	KX863339	*Holcocerus gloriosus*	Pakistan	Mapping global biodiversity connections with DNA barcodes: Lepidoptera of Pakistan	Muhammad A. et al., 2017	10 May 2012
MAMOT1710-12	KX862572	*Holcocerus gloriosus*	Pakistan	Mapping global biodiversity connections with DNA barcodes: Lepidoptera of Pakistan	Muhammad A. et al., 2017	8 May 2012
GBGL26655-19	MF071456	*Kerzhnerocossus tannuolus*	Russia	Review of the genus *Kerzhnerocossus* Yakovlev, 2011 (Lepidoptera: *Cossidae*) with descriptions of two new species from Russia and Mongolia	Saldaitis, A., 2011	\
GBGL26656-19	MF071457	*Kerzhnerocossus tannuolus*	Russia	Review of the genus *Kerzhnerocossus* Yakovlev, 2011 (Lepidoptera: *Cossidae*) with descriptions of two new species from Russia and Mongolia	Saldaitis, A., 2011	\
GBGL31256-19	KT713822	*Meharia semilactea*	\	Elusive ditrysian phylogeny: an account of combining systematized morphology with molecular data (Lepidoptera)	Heikkilae, M. et al., 2015	\
LEFIJ14982-20	\	*Meharia sp.*	Morocco	\	\	13 May 2010
GBGL31258-19	KT713823	*Meharia sp.*	\	Elusive ditrysian phylogeny: an account of combining systematized morphology with molecular data (Lepidoptera)	Heikkilae, M. et al., 2015	\
BCMI261-11		*Mormogystia proleuca*	Israel	iBOL Data Release	iBOL	27 May 2009
BCMI266-11		*Mormogystia proleuca*	Israel	iBOL Data Release	iBOL	31 May 2009
GWORZ172-10		*Parahypopta caestrum*	Italy	Species-Level Para- and Polyphyly in DNA Barcode Gene Trees: Strong Operational Bias in European Lepidoptera.	Mutanen, M. et al., 2016	23 June 1998
PHLAB331-10	HQ968493	*Phragmataecia castaneae*	Leichtenstein	Species-Level Para- and Polyphyly in DNA Barcode Gene Trees: Strong Operational Bias in European Lepidoptera.	Mutanen, M. et al., 2016	17 June 2009
CGUKA294-09	KX043172	*Phragmataecia castaneae*	UnitedKingdom	Species-Level Para- and Polyphyly in DNA Barcode Gene Trees: Strong Operational Bias in European Lepidoptera.	Mutanen, M. et al., 2016	17 July 2007
GWOR4145-09	KX071724	*Phragmataecia castaneae*	Germany	Species-Level Para- and Polyphyly in DNA Barcode Gene Trees: Strong Operational Bias in European Lepidoptera.	Mutanen, M. et al., 2016	8 June 2008
LEFIF226-10	HM874919	*Phragmataecia castaneae*	Finland	Testing DNA Barcode Performance in 1000 Species of European Lepidoptera: Large Geographic Distances Have Small Genetic Impacts	Huemer, P. et al., 2014	10 June 2002
GBGL13668-14	KC791461	*Streltzoviella insularis*	China	Forest Resource Conservation Department, Key Laboratory for Silviculture and Conservation of Ministry of Education	Li, J. & Chen, M.	28 July 2011
GBGL13669-14	KC791462	*Streltzoviella insularis*	China	Forest Resource Conservation Department, Key Laboratory for Silviculture and Conservation of Ministry of Education	Li, J. & Chen, M.	28 July 2012
GBGL13670-14	KC791463	*Streltzoviella insularis*	China	Forest Resource Conservation Department, Key Laboratory for Silviculture and Conservation of Ministry of Education	Li, J. & Chen, M.	28 July 2012
GBGL10959-12	JN673375	*Streltzoviella insularis*	Japan	RIKEN Plant Science Center, Advance NMR Metabomics Research Team	Vergara, F. et al.	\
GBMNC49243-20	MT785457	*Yakudza vicarius*	China	\	\	\
GBGL13671-14	KC791464	*Yakudza vicarius*	China	Forest Resource Conservation Department, Key Laboratory for Silviculture and Conservation of Ministry of Education	Li, J. & Chen, M.	28 July 2012
GBGL13676-14	KC791469	*Yakudza vicarius*	China	Forest Resource Conservation Department, Key Laboratory for Silviculture and Conservation of Ministry of Education	Li, J. & Chen, M.	28 July 2012
GBMNC49252-20	MF052057	*Yakudza vicarius*	China	Using full-length metabarcoding and DNA barcoding to infer community assembly for speciose taxonomic groups: a case study	Hao, M.D. et al., 2020	\
FBLMT329-09	HQ955163	*Zeuzera pyrina*	Germany	Species-Level Para- and Polyphyly in DNA Barcode Gene Trees: Strong Operational Bias in European Lepidoptera.	Mutanen, M. et al., 2016	24 July 1993
LEFIL178-10	JF854515.1	*Zeuzera pyrina*	Hungary	Species-Level Para- and Polyphyly in DNA Barcode Gene Trees: Strong Operational Bias in European Lepidoptera.	Mutanen, M. et al., 2016	16 June 2007
CGUKD508-09	KX043062.1	*Zeuzera pyrina*	United Kingdom	Species-Level Para- and Polyphyly in DNA Barcode Gene Trees: Strong Operational Bias in European Lepidoptera.	Mutanen, M. et al., 2016	18 July 2008
LON266-08	KX047718.1	*Zeuzera pyrina*	Norway	Species-Level Para- and Polyphyly in DNA Barcode Gene Trees: Strong Operational Bias in European Lepidoptera.	Mutanen, M. et al., 2016	26 July 2007
GU663065.1		*Noctua fimbriata*	France	iBOL Data Release	iBOL	13 July 1993

**Table A2:** Vegetation structure in ROSCI0295 Dealurile Clujului Est—Jucu de Sus (releves 1–4) and ROSCI0238 Suatu–Cojocna–Crairat–Cojocna (releve 5–6). Natura 2000 habitat: releve 1–4—6210 Semi-natural dry grasslands and scrubland facies on calcareous substrates (Festuco-Brometea); releves 5–6—6240* Sub-pannonan steppe grasslands.

			**No. of Releve**	1	2	3	4	5	6
			**Exposition**	W	W	W	W	S	S
									
			**Total Area (sq. m.)**	25	25	25	25	25	25
			**Total Cover (%)**	99	98	99	99	98	80
**U**	**T**	**R**	**Species**						
1.5	4	4	*Festuca rupicola* Heuffel	20	35	20	10	10	5
1.5	3.5	4	*Bromus japonicus* Thunb.	15		0.5	25	0.5	
				15			10		
3	0	0	*Achillea milleifolium* L.	10		2	10	2	2
1.5	3	3	*Xeranthemum cylindraceum* Sibth. et Sm.	10		0.5	15		
2	4.5	4	*Elymus hispidus* (Opiz) Melderis ssp. *hispidus*	5		20	15		
2	4	3	*Fragaria viridis* Weston	5		2	2		1
3	2	2	*Cruciata glabra* (L.) Ehrend.	5		0.5	0.5		
2.5	2.5	0	*Galium verum* L.	3	3	3	0.5	2	2
3	3	0	*Glechoma hederacea* L.	3					
1.5	3.5	4	*Thymus pannonicus* All.	2		0.5	0.5	2	
2.5	3	4	*Agrimonia eupatoria* L.	2	0.5	3	2	0.5	
3	3	0	*Inula britanica* L.	2		0.5	0.5		
2	3	5	*Medicago falcata* L.	1		0.5	0.5	0.5	1
				1	0.5	0.5	0.5		
2.5	3	3	*Clinopodium vulgare* L.	1	0.5	0.5	0.5		1
			*Torilis japonica* (Hoott.) DC.	1		0.5	0.5		
2.5	4	4	*Brachypodium pinnatum* (L.) Beauv.	0.5	5	15	0.5	2	2
2	3	4	*Euphorbia cyparissias* L.	0.5	5	5	0.5	0.5	3
2.5	3.5	3	*Crataegus monogyna* Jacq.	0.5		0.5	0.5	0.5	
3	3	0	*Odontites vernus* (Bellardi) Dumort.	0.5		0.5	0.5	0.5	
3	3	0	*Hypericum perforatum* L.	0.5	0.5	0.5		0.1	
2	3	4	*Artemisia absinthium* L.	0.5		2	0.5		
1	5	4	*Eryngium campestre* L.	0.5		0.5	1		
			*Daucus carota* L. ssp. *carota*	0.5		0.5	1		1
1.5	3	4	*Picris hieracioides* L.	0.5		0.5			
2.5	2.5	0	*Pimpinella saxifraga* L.	0.5	0.5	0.5	0.5		
1.5	3.5	4	*Potentilla recta* L.	0.5		0.5			
4.5	3	4	*Mentha longifolia* (L.) Hudson	0.5					
2	4	3.5	*Stipa tirsa* Steven		5			10	3
1	5	4	*Stipa capillata* L.					15	5
1	4.5	4.5	*Stipa lessingiana* Trin. et Rupr.					15	5
1	5	5	*Salvia nutans* L.					10	1
0	0	0	*Elymus repens* (L.) Gould.					5	2
2	4	4	*Teucrium chamaedrys* L.		0.5			5	1
2.5	3	4.5	*Filipendula vulgaris* Moench		5	5		3	1
2	5	4	*Dorycnium pentaphyllum* Scop. ssp. *herbaceum* (Vill.) Bonnier et Layens		15			3	
3	3	0	*Carex tomentosa* L.					3	3
2	3	4	*Phleum phleoides* (L.) Karsten					3	
1.5	3.5	4	*Inula ensifolia* L.					2	
2	4	4	*Thesium linophyllon* L.		0.5			2	
2	3.5	4	*Adonis vernalis* L.		3	0.5	0.5	1	1
1.5	4	4.5	*Cephalaria uralensis* (Murray) Roemer et Schultes					1	
1	4	5	*Teucrium montanum* L.					1	
3	0	4	*Dactylis glomerata* L.			7	0.5	0.5	5
2	3.5	4	*Bupleurum falcatum* L.		0.5	0.5		0.5	1
2	3	4	*Coronilla varia* L.			0.5	0.5	0.5	1
						0.5	0.5	0.5	
2	3	3	*Prunus spinosa* L.		0.5	0.5	0.5	0.5	
1.5	3.5	4	*Linum hirsutum* L.					0.5	
2	3.5	4.5	*Centaurea stoebe* L.					0.5	
								0.5	
1	5	4	*Ajuga laxmannii* (L.) Bentham		0.5			0.5	
2	4	4	*Odontites luteus* (L.) Clairv.					0.5	
2	4	4.5	*Onobrychis viciifolia* Scop.					0.5	1
2	4	4	*Echium maculatum* L.					0.5	1
1.5	5	3	*Dichanthium ischaemum* (L.) Roberty		5			0.5	1
2	4	4	*Thalictrum minus* L.		1			0.5	
2.5	0	4.5	*Plantago media* L.					0.5	1
2	4	4	*Galium glaucum* L.					0.5	1
1.5	4	4	*Astragalus monspessulanus* L.					0.5	
2	4	5	*Koeleria macrantha* (Ledeb.) Schultes ssp. *macrantha*					0.5	
2	3.5	4.5	*Potentilla arenaria* Borkh.					0.5	
2.5	3	5	*Salvia pratensis* L.					0.5	
2	4	4.5	*Crambe tatarica* Sebeok					0.5	1
2	3	5	*Asperula cynanchica* L.		0.5	0.5	0.5	0.1	1
3	4	4	*Juglans regia* L.					0.1	
1.5	4.5	4	*Plantago argentea* Chaix					0.1	
2.5	3.5	0	*Tragopogon dubius* Scop.					0.1	
1	4	4	*Veronica spicata* L. ssp. *spicata*		0.5			0.1	1
3.5	0	0	*Trifolium repens* L.			-	2		
2	3	0	*Poa angustifolia* L.			3	2		1
2.5	3.5	4	*Phlomis tuberosa* L.			2	0.5		15
2	3	3	*Origanum vulgare* L.			2			
2.5	4	3	*Salvia nemorosa* L.			2	0.5		10
4.5	3.5	4.5	*Iris sibirica* L.			0.5			
2	5	5	*Stachys recta* L.		0.5	0.5	0.1		3
2.5	3	3	*Inula salicina* L.		0.5	0.5			
1.5	4	4	*Stipa pennata* L.		10				
2	4	4	*Rosa gallica* L.		3				
3	3	4.5	*Prunella grandiflora* (L.) Scholler		0.5				
2	3.5	4	*Jurinea mollis* (L.) Reichenb. ssp. *transsylvanica* (Sprengel) Hayek						1
3	3	0	*Lolium perenne* L.						1
3	0	0	*Sonchus oleraceus* L.						1
									1
2	3	4.5	*Polygala major* Jacq.		0.5				
2	3.5	4	*Bromus pannonicus* Kummer et Sendtner						1
2	3	4	*Carex michelii* Host						2
3	3	0	*Cerinthe minor* L.						1
2.5	3.5	3.5	*Convolvulus arvensis* L.						2
2.5	0	0	*Lotus corniculatus* L.				0.5		1
3	0	0	*Plantago lanceolata* L.		0.5				
2.5	4	0	*Robinia pseudoacacia* L.						1
2	3.5	4	*Salvia austriaca* Jacq.		0.5				1
2	4	0	*Salvia verticillata* L.						5
2	3	3	*Seseli annuum* L.		0.5				
2.5	4	0	*Setaria pumila* (Poiret) Schultes						1
3	0	0	*Taraxacum officinale* Weber						1
3	2	3	*Tragopogon pratensis* L. ssp. *pratensis*						2

**Table A3:** The four CO1 sequences of *Paracossulus thrips*.

*>RONOC164-18 Paracossulus thrips* *AACATTATATTTTATTTTTGGAATTTGATCTGGAATAGTGGGTACTTCATTAAGTCTTTTAATCCGAGCTGAATTAGGAAACCCCGGATCATTAATTGGAAATGATCAAATCTATAACACTATCGTTACAGCTCATGCTTTTATTATAATTTTCTTCATAGTAATACCCATTATAATTGGGGGATTTGGTAATTGACTGGTTCCATTAATATTAGGAGCCCCTGATATGGCTTTCCCACGAATAAACAATATAAGATTTTGATTACTCCCCCCCTCATTAACCCTTTTAATCTCTAGAAGTATTGTTGAAAATGGAGCTGGCACAGGATGAACAGTTTATCCCCCATTATCTTCTAATATCGCTCATGGGGGTACTTCTGTTGACTTAGCAATTTTTTCCTTACATTTAGCAGGAATTTCCTCAATCCTAGGAGCTATTAATTTCATTACAACTATTATTAATATACGACCATACAACATATCATTTGATCAAATACCCCTATTTGTATGAGCAGTTGGAATTACTGCCCTATTATTACTTTTATCATTACCAGTATTAGCAGGAGCTATTACTATATTACTAACAGATCGAAATTTAAATACCTCATTCTTCGACCCAGCTGGAGGGGGAGATCCTATTTTATATCAACATTTATTT*
*>RONOC165-18 Paracossulus thrips* *AACATTATATTTTATTTTTGGAATTTGATCTGGAATAGTGGGTACTTCATTAAGTCTTTTAATCCGAGCTGAATTAGGAAACCCCGGATCATTAATTGGAAATGATCAAATCTATAACACTATCGTTACAGCTCATGCTTTTATTATAATTTTCTTCATAGTAATACCCATTATAATTGGAGGATTTGGTAATTGACTGGTTCCATTAATATTAGGAGCCCCTGATATGGCTTTCCCACGAATAAACAATATAAGATTTTGATTACTCCCCCCCTCATTAACCCTTTTAATCTCTAGAAGTATTGTTGAAAATGGAGCTGGCACAGGATGAACAGTTTATCCCCCATTATCTTCTAATATCGCTCATGGGGGTACTTCTGTTGACTTAGCAATTTTTTCCTTACATTTAGCAGGAATTTCCTCAATCCTAGGAGCTATTAATTTCATTACAACTATTATTAATATACGACCATACAACATATCATTTGATCAAATACCCCTATTTGTATGAGCAGTTGGAATTACTGCCCTATTATTACTTTTATCATTACCAGTATTAGCAGGAGCTATTACTATATTACTAACAGATCGAAATTTAAATACCTCATTCTTCGACCCAGCTGGAGGGGGAGATCCTATTTTATATCAACATTTATTT*
*>RONOC166-18 Paracossulus thrips* *AACATTATATTTTATTTTTGGAATTTGATCTGGAATAGTGGGTACTTCATTAAGTCTTTTAATCCGAGCTGAATTAGGAAACCCCGGATCATTAATTGGAAATGATCAAATCTATAACACTATCGTTACAGCTCATGCTTTTATTATAATTTTCTTCATAGTAATACCCATTATAATTGGAGGATTTGGTAATTGACTGGTTCCATTAATATTAGGAGCCCCTGATATGGCTTTCCCACGAATAAACAATATAAGATTTTGATTACTCCCCCCCTCATTAACCCTTTTAATCTCTAGAAGTATTGTTGAAAATGGAGCTGGCACAGGATGAACAGTTTATCCCCCATTATCTTCTAATATCGCTCATGGGGGTACTTCTGTTGACTTAGCAATTTTTTCCTTACATTTAGCAGGAATTTCCTCAATCCTAGGAGCTATTAATTTCATTACAACTATTATTAATATACGACCATACAACATATCATTTGATCAAATACCCCTATTTGTATGAGCAGTTGGAATTACTGCCCTATTATTACTTTTATCATTACCAGTATTAGCAGGAGCTATTACTATATTACTAACAGATCGAAATTTAAATACCTCATTCTTCGACCCAGCTGGAGGGGGAGATCCTATTTTATATCAACATTTATTT*
*>RONOC167-18 Paracossulus thrips* *AGCTCATGCTTTTATTATAATTTTCTTCATAGTAATACCNATTATAATTGGAGGATTTGGTAATTGACTGGTTCCATTAATATTAGGAGCNCCTGATATGGCTTTCCCACGAATAAACAATATAAGATTTTGATTACTNCCCCCCTCATTAACCCTTTTAATCTCTAGAAGTATTGTTGAAAATGGAGCNGGCACAGGATGAACAGTTTATCCCCCATTATCNTCTAATATCGCTCATGGGGGTACTTCTGTTGACTTNGCAATTTTTTCCTTACATTTAGCAGGAATTTCCTCAATCCTAGGAGCTATTAATTTCATTACAACTATTATTAATATACGACCNTACAACATATCATTTGATCAAATACCCCTATTTGTATGAGCAGTTGGAATTACTGCCCTATTATTACTTTTATCATTACCAGTATTAGCAGGAGCTATTACTATATTACTAACAGATCGAAATTTAAATACCTCATTCTTCGACCCAGCTGGAGGGGGAGATCCTATTTTATANCAACATTTATTT*
